# Tailoring of low grade coal to fluorescent nanocarbon structures and their potential as a glucose sensor

**DOI:** 10.1038/s41598-018-32371-9

**Published:** 2018-09-17

**Authors:** Manoj B, Ashlin M. Raj, George C. Thomas

**Affiliations:** Department of Physics & Electronics, CHRIST (Deemed to be University) Bengaluru, Karnataka, 560029 India

## Abstract

Lignite is an abundantly utilized feedstock for the facile synthesis of fluorescent carbon dots and carbon nanomaterials. Its value is appreciated as an energy source for combustion for long time. Herein we report a novel top-down strategy to synthesis lignite based fluorescent nano carbon structures by combined acidic oxidation and chemical reflux. The nanocarbon crystallites in lignite are converted to oxygenated nano carbon dots and graphene sheets. They exhibited stable fluorescence property in the visible region depending on their size, functionalities and defects which were highly stable in all the pH conditions. These nanocarbon structures are an effective probe for fluorescent sensing of label-free and selective detection of glucose ions with detection limit as low as 0.125 mM, promising real-world sensor applications. These findings establish a scalable method for the production of fluorescent carbon based glucose sensor from lignite.

## Introduction

Nanocarbon structures like graphene, graphene quantum dots (GQDs), carbon nanotubes (CNTs), carbon dots (CDs) and nanodiamonds have been extensively studied for their unique properties and applications. The fluorescence property and sensing potential of these materials are exceptionally high igniting research interest^1^. Coal, one of the inexpensive and abundant materials available, is a potential source for synthesizing carbon nanoparticles. The sp^2^ carbon domains embedded in coal matrix can be extracted by facile oxidation methods compared to other carbon precursors like graphite^2–4^.

Lignite being a low-grade coal has a large number of nanocarbon crystallites formed during the coalification process. One can develop a strategy to synthesize size-controlled carbon nanoparticles from lignite by a combination of oxidation, sonication and centrifugation techniques. The size and shape of the nano-crystallites present in coal depend heavily on coalification process and its maturity^5–7^. Fluorescence-based sensing has wide application in recent times owing to the notable properties such as exceptional sensitivity, low cost and short response time. Synthesis of fluorescent carbon dots (CDs) and graphene quantum dots (GQDs) have gained tremendous in recent years^1–4^. Coal-based carbon nanomaterial has higher sensitivity and extremely low metal ion detection limit compared to other conventional sensors^1,2^. The fluorescent property can be introduced to the coal nanoparticles by controlled oxidation and particle size reduction.

Diabetes is a group of metabolic disorder affecting more than 300 million people worldwide and the number keep increasing exponentially day by day. Monitoring the blood glucose level is imperative for diabetic patients. Electrochemical, spectroscopic and fluorescence properties of the sensors are some of the common techniques available for probing glucose molecules^8–11^. The currently available glucose sensors in the market use enzyme based electrochemical techniques for the quantification. Fluorescence based sensors have advantages of better sensitivity, short response time, low detection limit, making them better sensors for detecting hypoglycemia. Nevertheless, nanocarbon sensors could be significantly cheaper and more robust than the enzyme-based ones that are commonly available today. There are a few reported studies on fluorescence-based glucose sensors fabricated using carbon nanotubes and graphene oxides^8–11^. The production of these carbon nanostructures requires time and expensive synthesis equipment which escalate their cost.

This article reports the synthesis of cost-effective fluorescent carbon dots (CDs) and graphene oxide layers from lignite using facile techniques. These highly stable fluorescent CDs have high potential in sensing glucose molecule with a detection limit of 0.125 mM making them an ideal nanocarbon based glucose sensor. To the best of our knowledge, this is the first reported study on glucose detection with the aid of lignite based nanocarbon.

## Results and Discussion

The nano carbon structures are extracted from lignite by simple chemical methods. The obtained nanocarbon fractions are refluxed with CHCl_3_ several times to promote de-agglomeration and are designated as LC1C (Residue), LC2C (Supernatant) and dialysate (LC3C) (Explained in Materials and Methods). Elemental analysis (CHNS analysis-Table 1 and Supplementary Information) of the samples reveal that they consist of organic structures with Carbon (C), Oxygen (O), Nitrogen (N) and Hydrogen (H) as major elements and small quantities of Sulfur (S) as a minor element which are inherent in any organic solid fuel. The carbon content is found to be varying from 47.07, 34.83 and 39.73 wt% for the samples LC1C, LC2C and LC3C respectively. The high oxygen content makes the synthesized nano material to oxygenated carbon structures. Surprisingly the carbon structures obtained after refluxing with the nitric acid (LS200) - (Refer Materials and Methods) are highly oxygenated nanocarbon sheets (O = 79.84 wt% while C = 10.70 wt%). The presence of Sulfur is also marginally increased compared to other samples.

**Table 1:** Elemental analysis of nanocarbon structures from lignite.

Sample	N (wt.%)	C (wt.%)	S (wt.%)	H (wt.%)	O (wt.%)
LC1C	2.60	47.07	1.41	5.07	43.85
LC2C	2.41	34.83	1.32	2.51	58.93
LC3C	3.25	39.73	1.49	4.01	51.52
LS200	2.89	10.70	4.69	1.88	79.84

**Figure 1:**
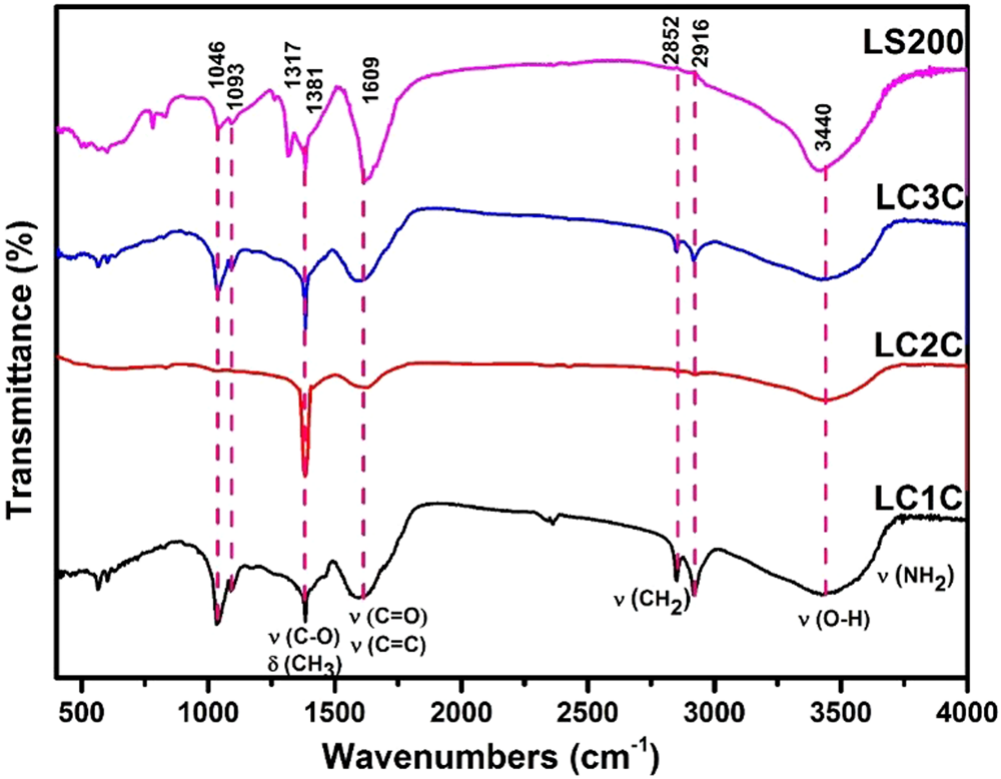
FTIR spectra of carbon nano structures.

The chemical structure was further confirmed by FTIR (Fig. 1), XPS and Raman Spectra (Refer Supporting information Figs S1 and S2). The spectra of LC1C and LC3C exhibit comparable characteristics. The band at 1600–1620 cm^−1^ is originated from the conjugated C=C stretching vibration & carbonyl groups present in the carbon dots. The 1381 cm^−1^ peak is due to the presence of cyclic carbon groups. The band at 2922 cm^−1^ arises from the asymmetric vibration of sp^3^ bond in CH_2_ and CH_3_ groups in the hydrocarbon network^11–14^. The peak at 2852 cm^−1^ is ascribed to the overlapping of symmetric vibration of the sp^3^ band for CH_3_ and CH_2_ functional groups. The 3442 cm^−1^ peak arises from the presence of OH group of alcohols, carboxylic groups and absorbed water. In the spectra of LC2C, many functional groups except the carbonyl and carboxyl groups are absent. In the LS200 sample, one could see an abundance of functional groups especially C-O and C=O with the C=C band.

The FT-IR analysis confirms that the nano carbon contains abundant graphitic structure with hydroxyl and carbonyl functional groups^11^. This result is in support of the elemental analysis result (Table 1). These nanocarbons are highly soluble and form a stable suspension in water owing to the oxygen functional groups.

The XPS analysis of the synthesized nanostructure from Lignite is obtained and presented in supporting information Fig. S1. C1s XPS spectra clearly indicate that they have been functionalized by oxygen moieties forming four different species namely, the sp^2^ Carbon (~284 eV), C in C-O bonds, C bonded to O as epoxy/hydroxyl (~286 eV), Carbonyl C, C=O of alcohols/phenols/ether (~287.1 eV) and the carboxylate C, O-C=O (~288.7.0 eV)^15^. The XPS results are well corroborated by the FTIR spectra, which shows the presence of carbonyl, carboxyl, epoxy and hydroxyl functional groups in all the nanostructures.

Raman spectra of the original precursor (Fig. S2) shows two broad and strongly overlapping D and G bands at around 1347 cm^−1^ and 1583 cm^−1^ respectively, similar to that of turbostratic graphitic nanocarbon. The D-band occurs as a result of defects and also due to the broken symmetry of basal plane of the graphitic carbon atoms^16,17^. The G-band corresponds to the E_g_ vibrational mode of sp^2^ hybridized carbon atoms in both the rings and chain structures. The 2D region consists of bumps, implying the formation of few graphene layers with wrinkles and defects. In the Raman spectra, the relative D to G band intensity (I_D_/I_G_) is associated with the size and physical or chemical defects present in the crystallographic plane of the characteristic graphene oxide sheets. The I_D_/I_G_ ratio is close to unity indicating the presence of defects in the nanoparticles.

**Figure 2:**
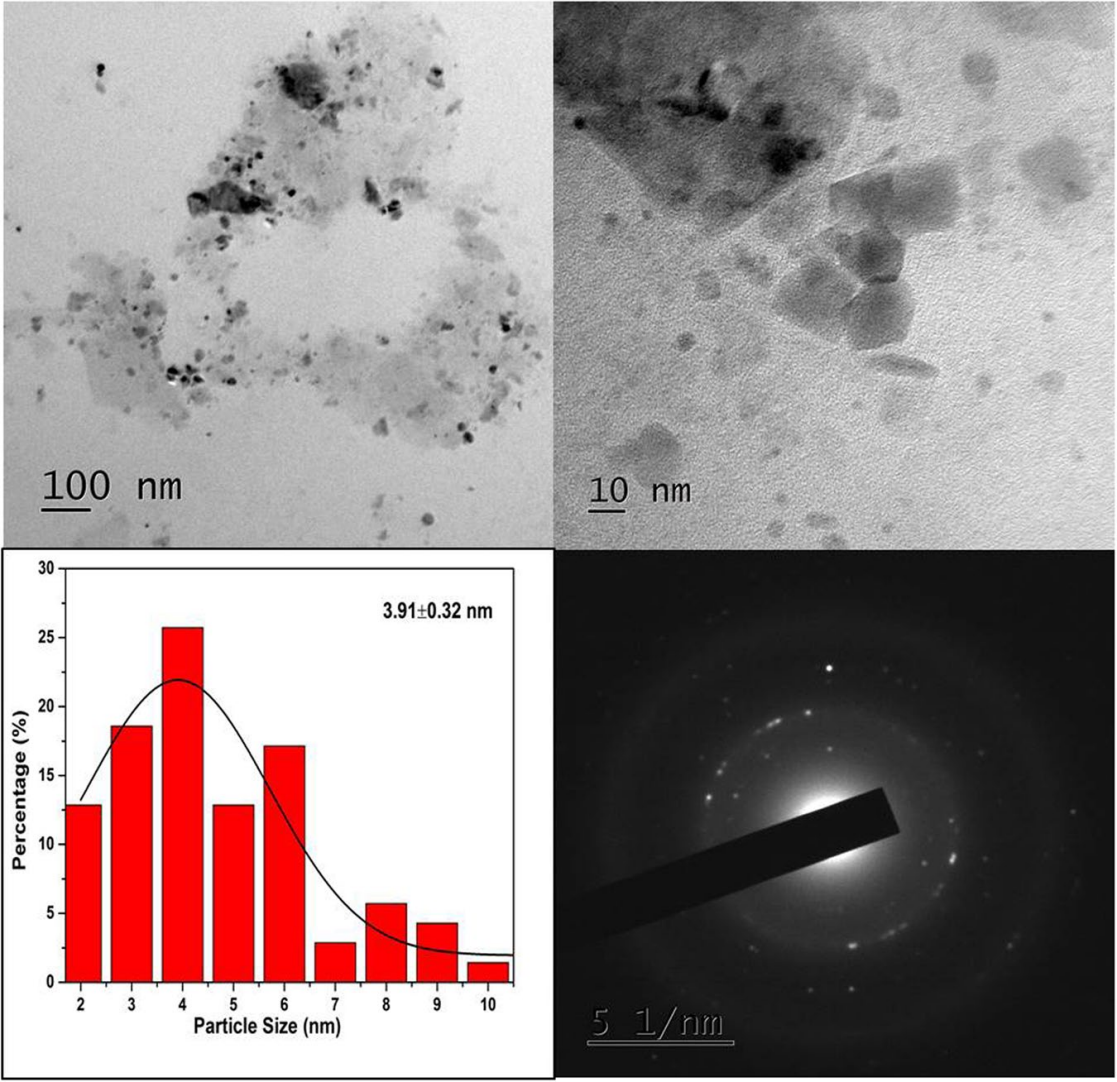
TEM image of LC1C nano dots (The particle size is in the range 3–4 nm).

**Figure 3:**
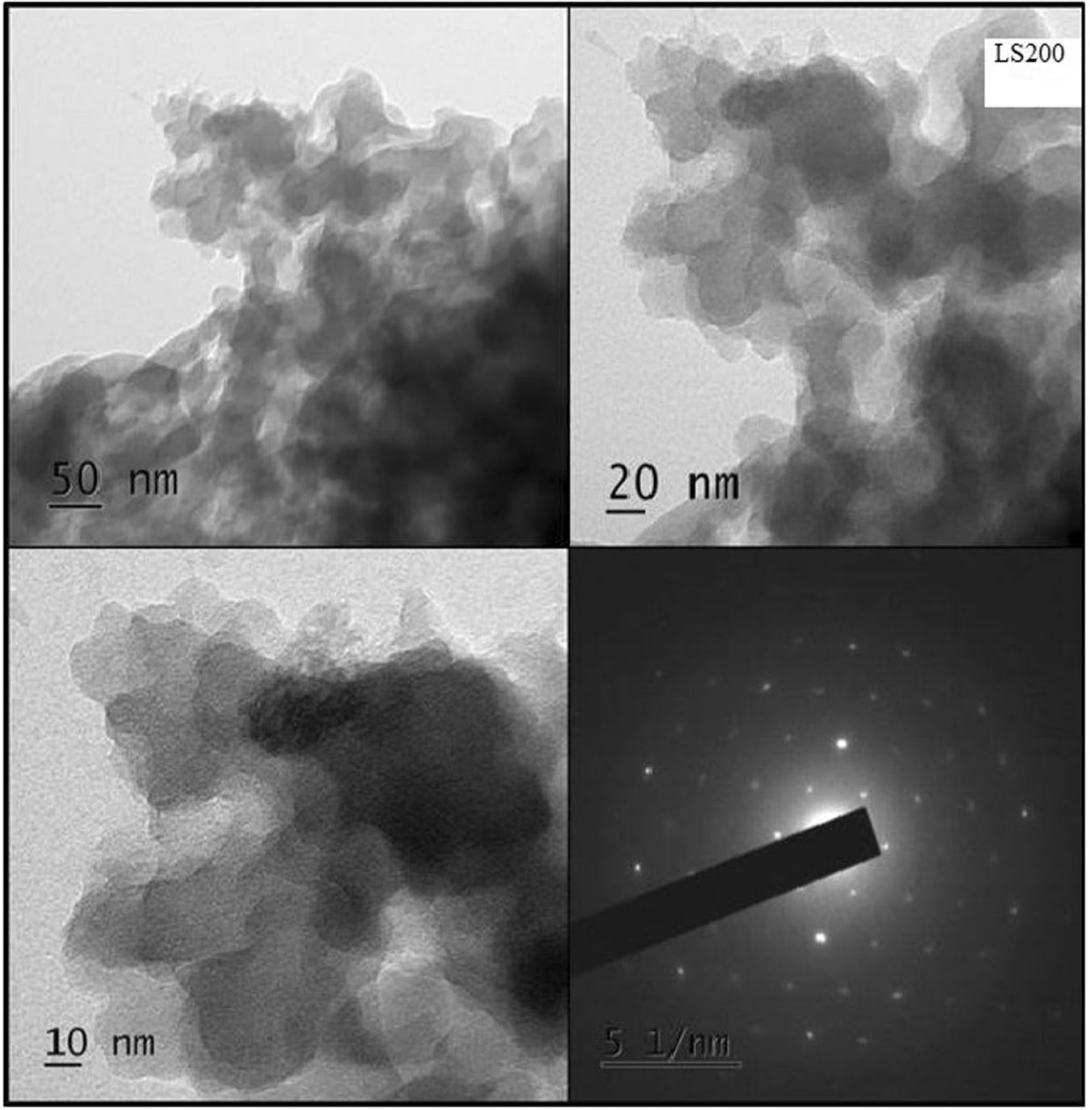
TEM image of LS200 exhibiting formation of graphene layers.

The surface morphology of the nanostructure is further analyzed by transmission electron microscope (TEM) (Figs 2 and 3) TEM image of LC1C (Fig. 2) shows nanodots with circular, rectangular and hexagonal shapes with majority of particles in the size range 3–4 nm. The lattice spacing is measured to be 0.51 nm (Supplementary information Fig. S3), owing to the incorporation of oxygen to the crystalline plane of graphene^1,18,19^. The SAED pattern shows the nanocrystalline nature of the nanodots obtained. The TEM analysis of the nanostructure (LC2C) is presented in the Supplementary Fig. S4. The formation of a twinning plane is observed in the TEM image^20^. One could observe heterogeneous structures due to stacking of the graphene oxide layers. The selected area electron diffraction (SAED) pattern show rings corresponding to the (111) and (200) planes of graphite^4^. The TEM image of the sample LC3C is embedded with nanodots into the carbon matrix (Refer Supplementary Fig. S5). The corresponding SAED pattern shows ring pattern which can be attributed to the formation of poly nanocrystalline carbon^1^.

The TEM image of the nitric acid refluxed product (LS200) is presented in Fig. 3. In the micrograph, darker region is due to stacking of multilayers while lighter region is attributed to formation of few-layer graphene oxide^4^. The SAED pattern of the sample confirms the hexagonal atomic structure and high crystalline nature of the sheets^1,4^.

The TEM anlysis of LC1C sample confirms the presence of carbon nanoparticles with size in the range of 3–4 nm. Stacking and agglomeration are observed in the LC2C and LC3C nanostructures. This agglomeration is either caused by surface dangling bonds of the carbon dots or in the process to attain stability by surface atoms with high energy. This effect is highly dependent on the preparation method, more specifically, the solvent around, the temperature of the sample formation, pH conditions as well as other external actions such as mechanical stress and electric field. Formation of few layer graphene oxide with high crystalline nature is noticed in LS200. Raman studies confirm the presence of defects in oxygenated nanocarbon structures. The XPS and FTIR spectra reveal the presence of oxygen and carbonyl functional groups in all the samples. It is inferred that one could tailor the size and shape of preformed nanocarbon crystallites in lignite to oxygenated carbon dots and graphene oxide layers by simple chemical methods.

### Optical properties of as-synthesized carbon nanoparticle

**Figure 4:**
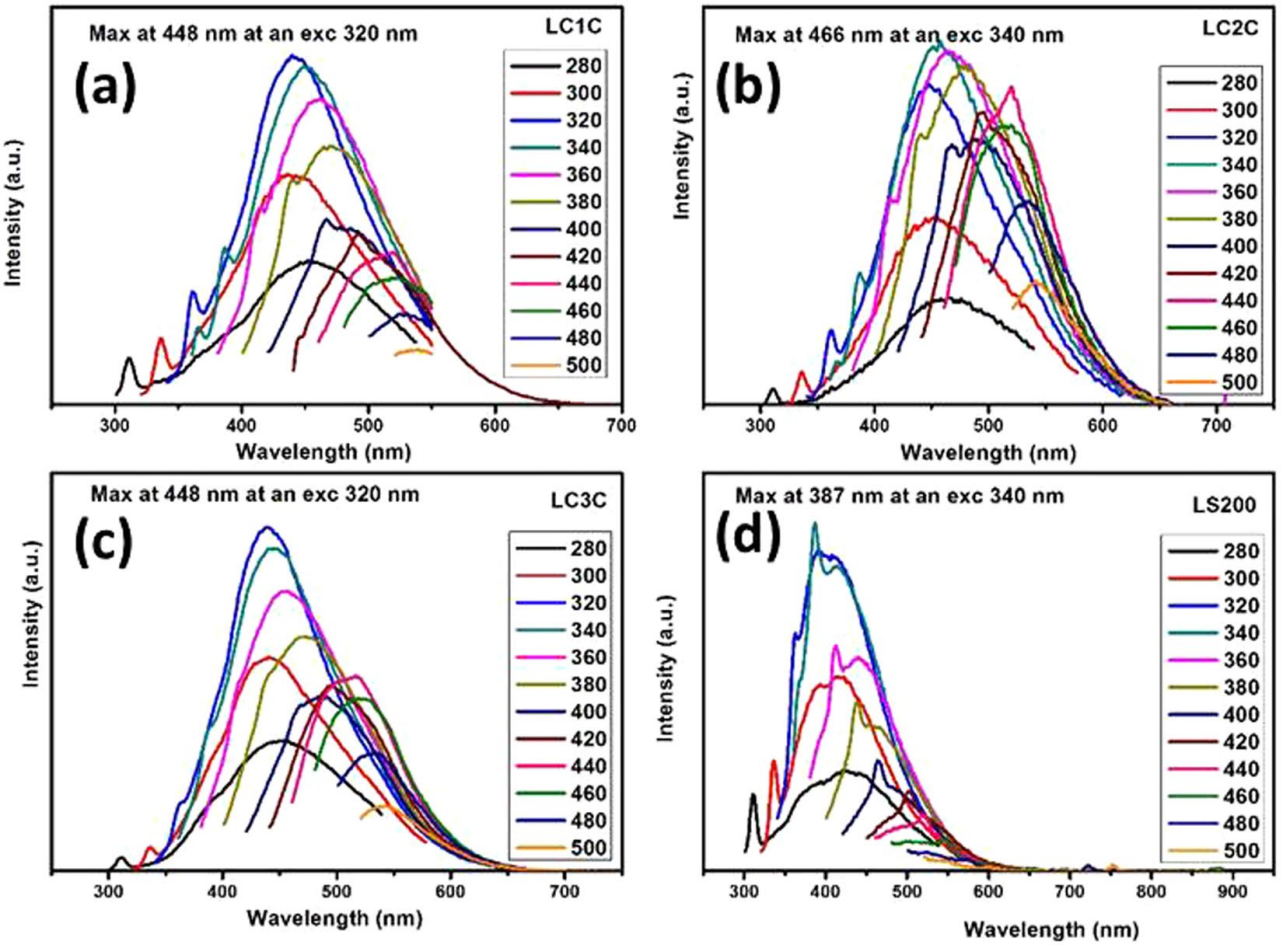
(**a–d)** Photoluminescence emission of as-synthesized carbon nanoparticles under multiple excitations (**a**) LC1C (**b**) LC2C (**c**) LC3C (**d**) LS200.

The optical properties of the CDs are investigated by the Fluorescence analysis (Refer Fig. 4(a–d)) and UV analysis (See Table 2 and Supplementary information Fig. S6).

**Table 2:** Optical bandgap of the synthesized nanocarbon structures.

Samples	Energy gap (eV)
LC1C	2.90
LC2C	2.50
LC3C	3.40
LS200	2.70

The variation in the energy gap of the nanostructure is determined from the UV-Visible analysis by employing Tauc Plot^21^. The energy gap value is found to be in the range 3.40 eV to 2.50 eV, which is in the reported range of organic semiconducting dots^1^. Out of all the nanoparticles, LC2C possess the smallest energy gap of the order of 2.50 eV and LC3C has the band energy of 3.40 eV.

**Figure 5:**
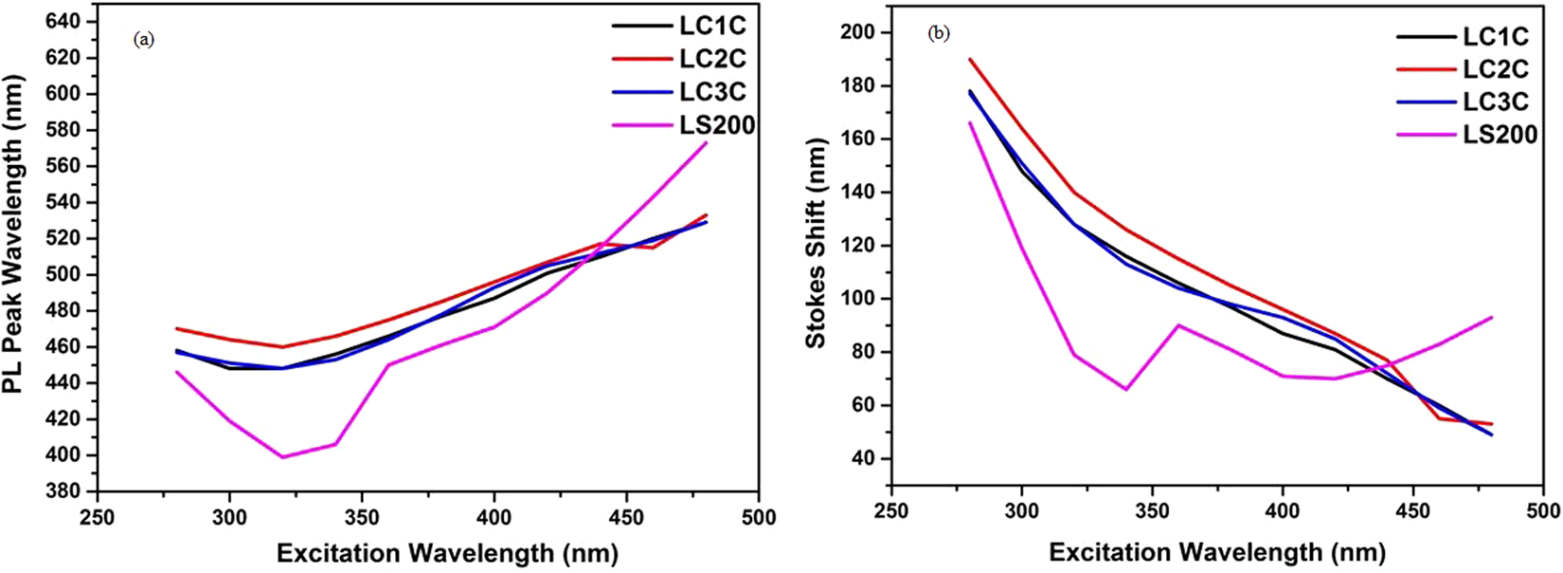
(**a**) Change in photoluminescence (PL) emission peak wavelength vs Excitation wavelength (**b**) Stokes shift vs Excitation wavelength.

The fluorescence spectra of the oxygenated carbon dots are recorded by varying the excitation wavelength from 280 to 500 nm with an interval of 20 nm. The fluorasense spectra of all samples exhibit a redshift with the excitation wavelength. At higher wavelngth surface state emission dominates and contribute to the excitation dependent behavior^1^. LC1C and LC3C shows maximum emission at an excitation of 320 nm while LC2C and LS200 exhibit maximum emission at an excitation of 340 nm. Change in photoluminescence (PL) emission peak wavelength and Stokes shift vs Excitation wavelength for the nanoparticles are presented in Fig. 5(a,b).

All the carbon structures show a hypsochromic shift initially till 320 nm and followed by a bathochromic shift. The Stokes shift, originates due to the difference between the position of the emission maxima and the excitation spectra, is an indicator to confirm the quantum confinement effect in nanocrystals^1^. If the luminescence mechanism is affected by the size distribution, the stokes shift approaches linearly to zero with increasing excitation wavelength^1^. This confirms the existence of band gap in the nano carbon material^22–26^. The excitation wavelength vs stokes shifts plot (Fig. 5b) gives a response trending to zero for LC1C and LC3C, which confirms the particle size effect as one of the reasons for fluorescence. This trend is not observed for other two nano carbon samples indicating that carbogenic core doesn’t play any role in the fluorescence property. The fluorescence in the nanoparticles is mainly due to the combination of quantum size effect, oxygen functional groups and defect states^1,27^ (as evident from the FTIR, XPS and Raman Analysis).

### Influence of pH on the PL of CDs

**Figure 6:**
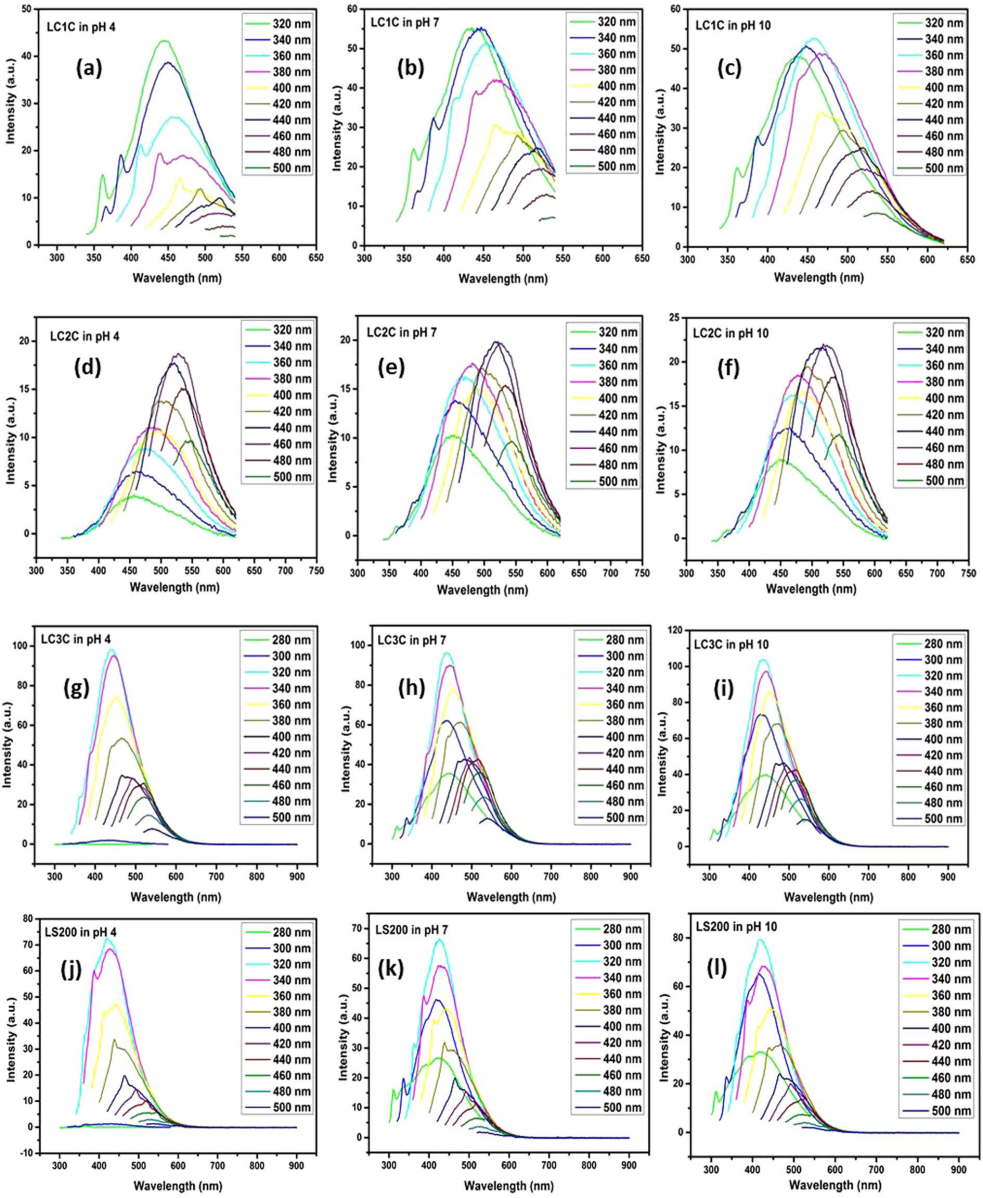
(**a**–**l**) The Fluorescence response of carbon nanoparticles LC1C, LC2C, LC3C and LS200 under acidic pH, neutral pH and alkaline conditions.

Figure 6(a–l) shows the fluorescence response of the synthesized nanostructures under diffferent excitation wavelengths in acidic, basic and neutral pH conditions. The nanocarbons are dissolved in a buffer solution of three different pH (acidic-4, neutral-7 and alkaline-10) and their excitation dependent PL is studied by changing the excitation wavelength from 320 nm to 500 nm in steps of 20 nm. The results are presented in Fig. 6(a–l). It is noticed that all the samples behaves distinctly from one another in their fluoresence behavior. Fluorescene emission peak of LC1C & LC3C is found to be centred around 448 nm at an excitation wavelength of 320 nm at all pH. The LC2C & LS200 has its emission maxima centred around 515 nm when excited at 340 nm. The sample do not show any significant variation in position and intensity of its fluorescence spectra with change in pH of the solvent. Change in pH value also induces a change in the agglomeration^28^, but from Fig. 4(a–i), it is understood that even when the agglomeration level is different at different pH, the fluorescence peak position of the samples has not shifted. The pH independent fluorescence spectra reveals that the carbon nanostructures are stable in different environment.

### Detection of glucose

Sensors continue to have a great impact on everyday life. Production of highly selective, sensitive and cost-effective sensors are in very high demand. Nanocarbon based materials have the quoted advantage of high surface area, stability, good conductivity and great biocompatibility. These properties make them suitable for making biocompatible, fluorescent electrochemical sensors^29,30^.

Graphene produced by chemical methods have a large number of oxygen-containing functional groups like carboxyl, epoxy and hydroxyl groups on the graphene surface. This makes them hydrophilic and dissolvable in water or organic solvents^3,8^. These functional groups act as an active site for the glucose molecules which have a significant effect on the PL property of the CDs.

**Figure 7:**
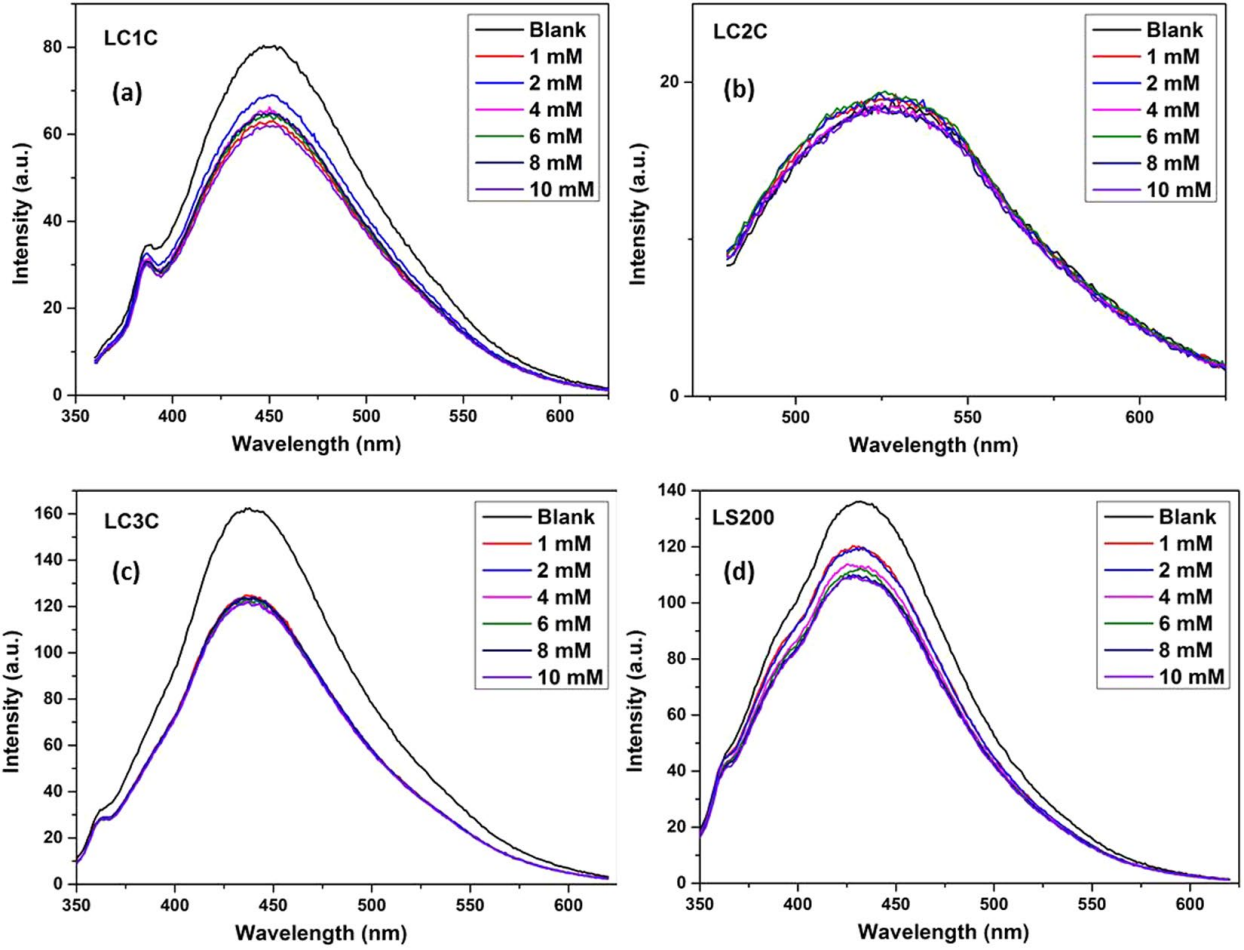
(**a–d**). Fluorescence quenching of nanostructures with glucose of various concentration.

To evaluate the potential of synthesized nanocarbon in detecting the glucose ions, fluoresence quenching with varying concentration is carried out in neutral pH. (Refer UV-Visible analysis -Fig. S7 and fluorescence analysis (Fig. 7(a–d) and Supplementary Figs S8 and S9)). To the nanoparticles (LC1C, LC2C, LC3C and LS200 separately), various concentrations of glucose solution (10 mM to 1 mM) were added and shaken for uniform mixing. The UV Visible spectra analysis shows very good quenching of the absorption peak with the addition of glucose to the nanoparticle (Fig. S7). LC1C and LS200 exhibit maximum quenching of the absorption peak. To identify the limit of sensing and efficiency, fluorescence spectra analysis is carried out at an excitation of 320 nm for LC1C and LC3C while LS200 & LC2C are excited at 340 nm in the presence of different concentration of glucose. It is noticed that the value F/F_o_ (ratio of fluorescence intensity of the probe with and without glucose ions) of the samples decreases as the concentration of glucose ion changes from 1 mM to 10 mM (Fig. 7(a–d)). The fluorescence quenching may be attributed to the following reasons. LC1C being carbon dots of wide size range and shape has large surface area. The oxygen functional groups are attached to the edges of these nanodots producing the fluorescence spectra. The added glucose molecule gets attached to these oxygen functional groups leading to quenching of the fluorescence spectra. As the concentration of glucose increases, the quenching of the fluorescence spectra also exhibit a linear trend from 1 mM to 10 mM range. A similar linear quenching is also exhibited by the LS200 graphene oxide layers (Supplementary Figs S8 and S9). The oxygen functional groups are attached to the edges of the graphene oxide layer. When glucose molecules are added, it gets attached to oxygen functional groups, resulting in quenching of the fluorescence spectra. This sample (LS200) could detect glucose level as low as 0.125 mM (presented in Supplementary information Fig. S10). The LC2C and LC3C sample shows nonlinear quenching in the presence of glucose (Fig. S9). This may be due to the fact that the oxygen functional are in the basal plane of the stacked structures and are not freely available for bonding with glucose molecule.

In summary, we report a strategy for synthesizing size controlled fluorescent nanocarbon from lignite, an abundant and ubiquitous natural carbon feedstock. The size and fluorescence property of lignite based carbon nanodots can be tuned by altering the structure of preformed carbon crystallites. The fluorescence in the nanoparticles is mainly attributed to the combination of quantum size effect, oxygen functional groups and defect states. They exhibit pH independent luminescence in aquoeous solutions without any passivation agent. The synthesized carbon nanostuctures LC1C and LS200 shows good linear fluorescence quenching with addition of glucose molecule mainly due to larger surface area of the carbon dots and oxygen functional groups attached to it. It is worthwhile to mention that these nanocarbon structures are a promising glucose detection probe for hyperglycemia with detection limit as low as 0.125 mM. This finding could lead to the development of a low cost glucose sensor using low grade coal as precursor.

## Materials and Methods

### Synthesis of nanocarbon structures from lignite

The preformed carbon nanostructures (GQDs and CDs) were extracted from lignite by ultrasonicating the lignite powder with dil.HNO_3_ followed by stirring for 1 hour at 100 °C in a magnetic stirrer. The mixture was neutralized with sodium hydroxide. The resultant suspension was centrifuged (at 11500 rpm) for one hour to separate the supernatant and residue. The residue solution was dried at 80 °C for 2 days in a vacuum furnace. The supernatant was filtered through a dialysis membrane which retained the larger particles (LC2) and the dialysate is retained as LC3. The products were further reflexed with chloroform (CHCl_3_) several times and powder was obtained after drying in vacuum to obtain the product LC1C, LC2C and LC3C. Alternatively, about 5 g of lignite sample was crushed into a fine powder using mortar and pestle. 2 g of the pulverized sample was added to concentrated nitric acid (70%) and stirred for 24 hours with a magnetic stirrer. The mixture was centrifuged at 12000 rpm for an hour to separate the residue and supernatant. The supernatant was heated in a vacuum oven at 200 °C to obtain a yellow powder (LS200).

### Characterization

Raman measurements were performed at an excitation wavelength of 514 nm using Horiba LAB AM-HR spectrometer. The compositional analyses of the samples were carried out using X-ray photoelectron spectroscopy (XPS Omicron ESCA probe with monochromatic (Al) X-radiation. TEM analyses of the samples were done using a JEOL JEM-2100 model. The fluorescent study was carried out with an RF-5301 PC, Shimadzu fluorescence spectrometer. Functional groups were qualitatively identified by a Fourier transform infrared spectrometer (Thermo Nicolet 370 spectrophotometer). The UV/Vis spectrometer (Ocean optics JAZ series) was used for acquiring absorption spectra. The CHNS analysis was carried out on the instrument Elemental Vario EL111 elemental analyzer.

## Electronic supplementary material

Supplementary Information
