# Supplementary material for: Tailoring of low grade coal to fluorescent nanocarbon structures and their potential as a glucose sensor
Source: Sci Rep. 2018 Sep 17;8:13891. doi: 10.1038/s41598-018-32371-9 (PMC6141539; doi:10.1038/s41598-018-32371-9)
Supplement: Supplementary file 1 — Supplementary Information [file 41598_2018_32371_MOESM1_ESM.pdf]

Tailoring of low grade coal to fluorescent nanocarbon structures and their potential as a glucose sensor

MANOJ B, ASHLIN M RAJ, GEORGE THOMAS C

Department of Physics & Electronics, CHRSIT (Deemed to be University) Bengaluru, Karnataka, India, 560029

E-mail: manoj.b@christuniversity.in/

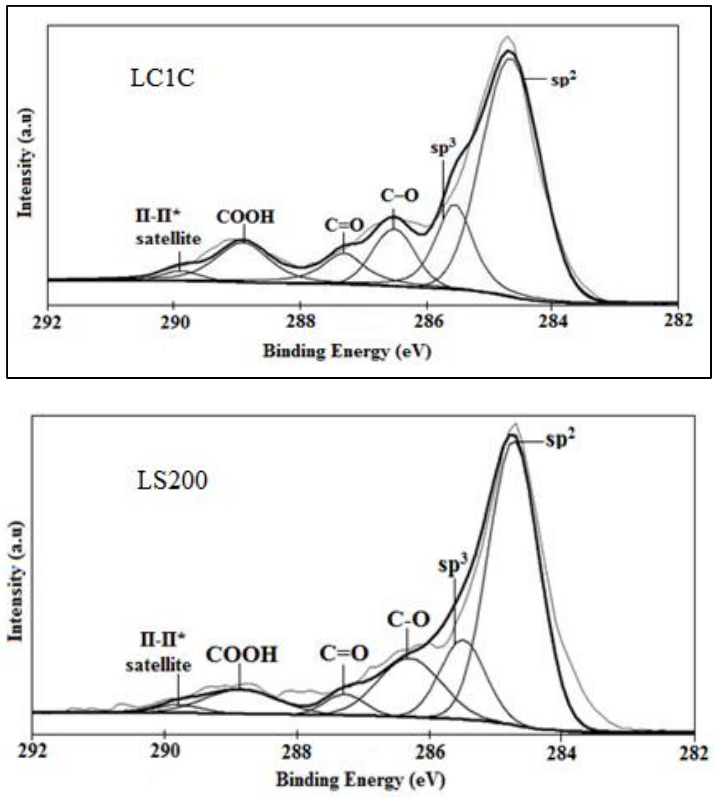

Figure S1. Deconvoluted C1s XPS spectra of nano carbon structures from lignite

1s XPS spectra of the carbon nanostructures clearly indicate that they have been functionalized by oxygen moieties forming four different species namely, the sp^2 Carbon (~284 eV), C in C-O bonds, C bonded to O as epoxy/hydroxyl (~286 eV), Carbonyl C, (i-e) C=O of alcohols/ phenols/ether (~287.1 eV) and the carboxylate C, O-C=O (~288.7.0 eV).)

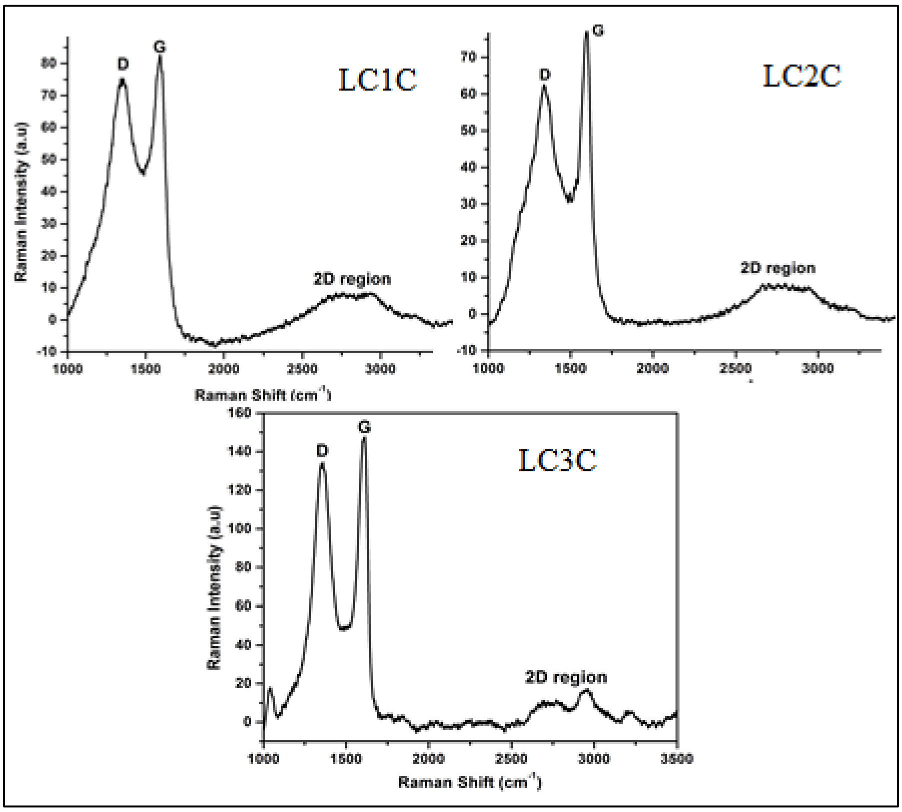

Figure S2. Raman Analysis of the nanocarbon structures from lignite

(The D-band occurs as a result of defects and also due to broken symmetry of basal plane of the graphitic carbon atoms. The G-band corresponds to the E_g vibrational mode of sp^2 hybridized carbon atoms in both the rings and chain structures. A broad 2D band is also observed at around 2750 cm^{-1} implying layered graphitic structure)

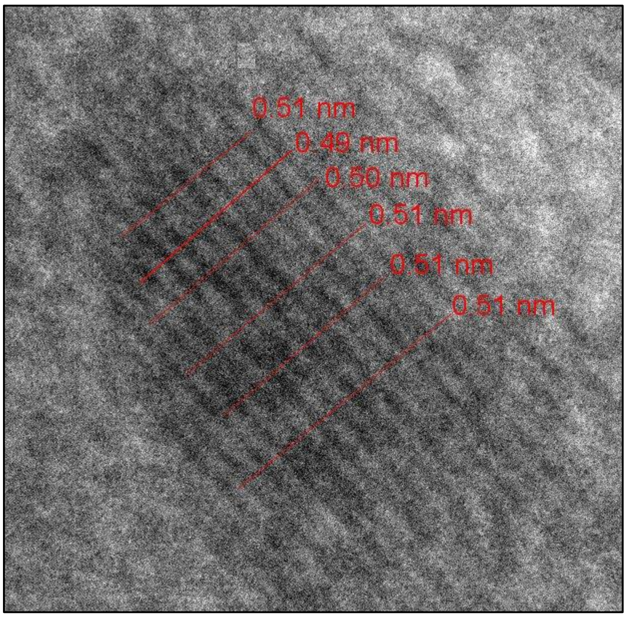

Figure S3. Lattice spacing of the LC1C nanostructure

Lattice spacing in LC1C nanostructure is of the order of 0.51 nm.

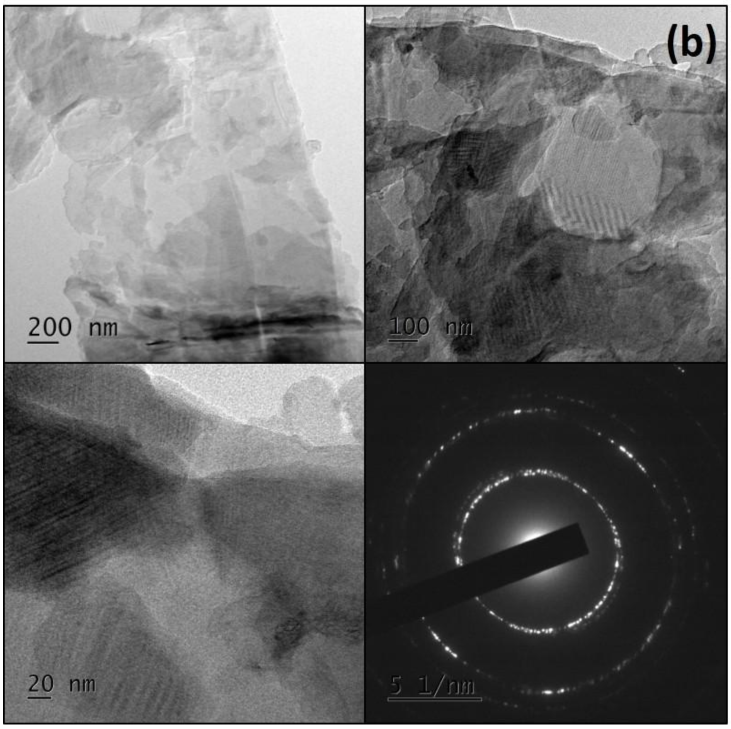

Figure S4. TEM image of LC2C nanostructure exhibiting formation of twinning planes and stacking

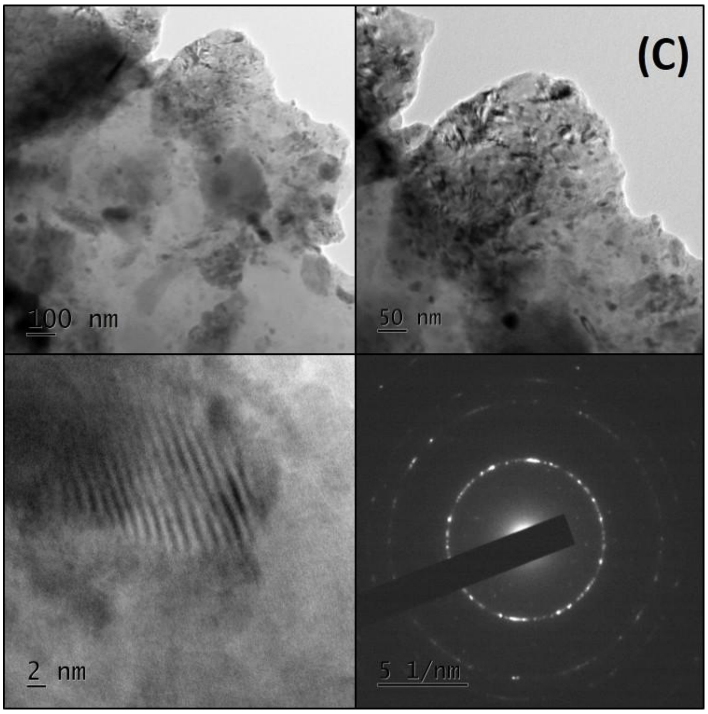

Figure S5. TEM image of LC3C nanostructure exhibiting formation of stacking carbon dots and layers

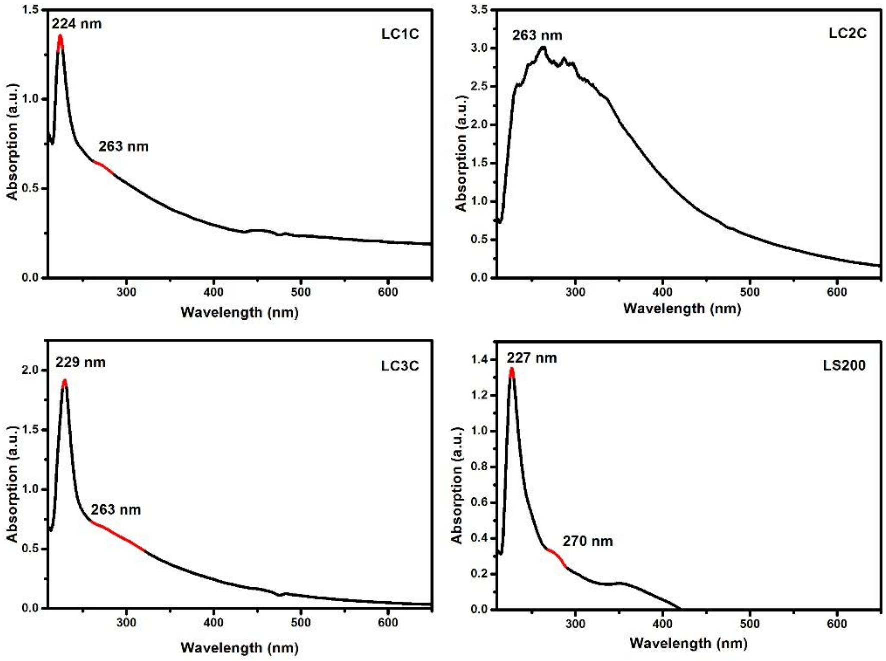

Fig S6. UV-Visible Spectra of the nano carbon structures from lignite

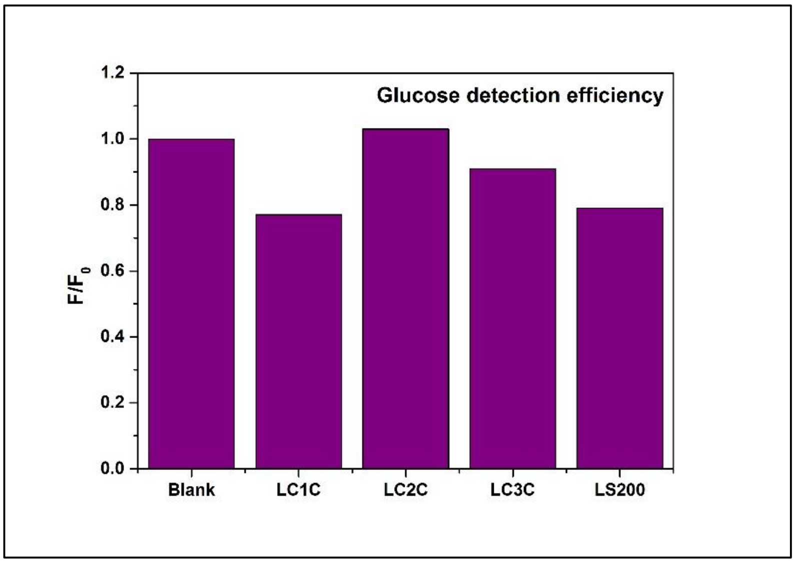

Fig. S7. Comparison of UV absorption intensity of various nanocarbon samples with and without glucose

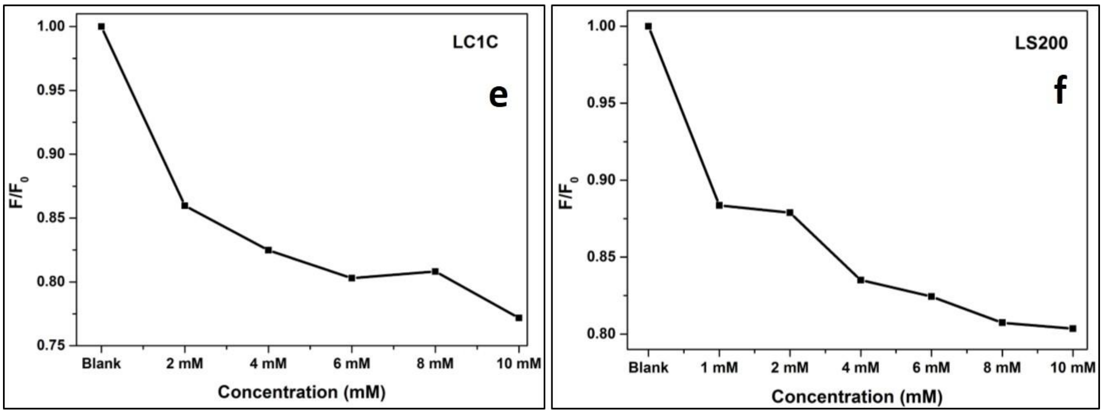

Fig S8. Fluorescence quenching of (e) LC2C and (f) LC3C with glucose of various concentration (Good degree of linearity in quenching is noticed)

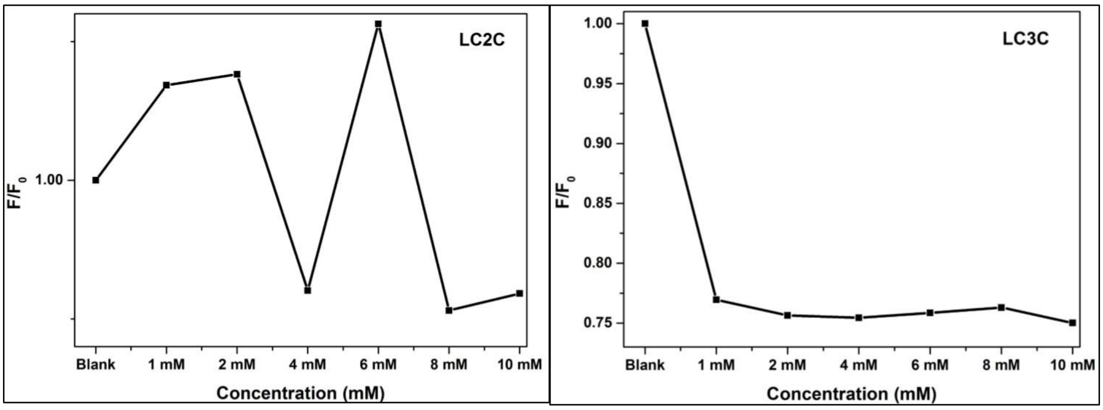

Fig S9. Fluorescence quenching of LC2C and LC3C with glucose of various concentration

(Irregular fluorescence quenching of LC2C and high quenching of LC3C nanostructure in the presence of glucose molecule)

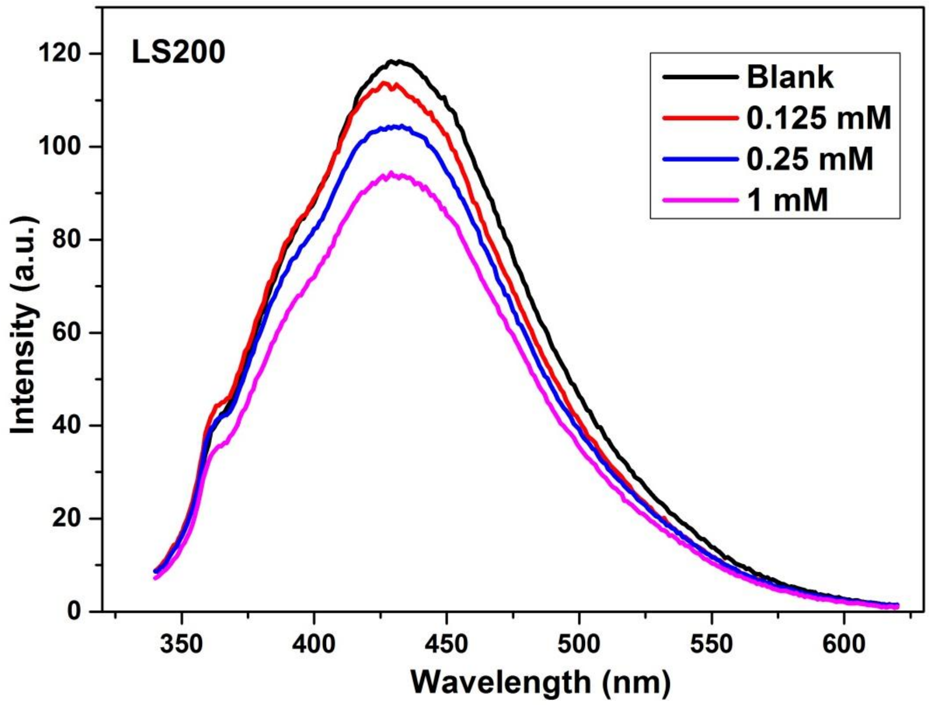

Fig S10.. Fluorescence quenching of the LS200 nanocarbon with the addition of low concentration of glucose (Indicating systematic quenching with concentration range of 1 mM to 0.125 mM).

Characterization techniques: Elemental Analysis (CHNS Analysis)

Ultimate analysis is an accurate elemental analysis technique which precisely estimates the percentage of elements present in organic matrices and other types of materials in coal. The Ultimate Analysis (CHNS analysis) was carried out on the instrument Elemental Vario EL111 elemental analyzer.

Sample Name	N% (wt%)	C%	S%	H%	Weight, mg
**LCIC**	2.60	47.07	1.41	5.07	2.84
**LC2C**	2.41	34.83	1.32	2.51	2.95
**LC3C**	3.25	39.73	1.49	4.01	2.91
**LS200**	2.89	10.70	4.69	1.88	3.02

CHNS analysis is based on the principle of "Dumas method", which involves complete and instantaneous oxidation of coal by "flash combustion". The products are separated by a chromatographic column and detected by a thermal conductivity detector (TCD), whose output signal is proportional to the concentration of individual components in the mixture. The sample weighed in milligrams (in the present study we have taken 2.84 mg-3.02 mg as shown in table) housed in a tin capsule is dropped into a quartz tube at 1020˚C with constant helium flow (carrier gas). Prior to this experiment, the stream is enriched with measured amount of high

purity oxygen to achieve a strong oxidizing environment, ensuring complete combustion of thermally resistant substances. The gas mixture is initially driven through an oxidation catalyst (WO_3) zone and later a copper zone. The reduction of nitrogen oxides and sulphuric anhydride (SO_3) leads to elemental nitrogen and sulphurous anhydride (SO_2), retaining excess oxygen. The gases are then passed through the absorbent traps and leave only carbon dioxide, water, nitrogen and sulphur dioxide, which are detected by a thermal conductivity detector. While measuring oxygen, the sample undergoes instant pyrolysis in a stream of helium gas, confirming conversion of organic oxygen into carbon monoxide separated on a gas chromatographic (GC) column packed with molecular sieves.

From ultimate analysis, the oxygen is estimated by deducing from 100, the sum of the percentages of moisture, ash, carbon, hydrogen, nitrogen and sulphur (ASTM D-3176).
